# A comprehensive portrait of Y-STR diversity of Indian populations and comparison with 129 worldwide populations

**DOI:** 10.1038/s41598-018-33714-2

**Published:** 2018-10-18

**Authors:** Mugdha Singh, Anujit Sarkar, Madhusudan R. Nandineni

**Affiliations:** 1 Laboratory of Genomics and Profiling Applications, Centre for DNA Fingerprinting and Diagnostics, Uppal, Hyderabad, Telangana State 500039 India; 2 Graduate studies, Manipal Academy of Higher Education, Manipal, Karnataka India; 3 Laboratory of DNA Fingerprinting Services, Centre for DNA Fingerprinting and Diagnostics, Uppal, Hyderabad, Telangana State 500039 India

## Abstract

India, known for its rich cultural, linguistic and ethnic diversity, has attracted the attention of population geneticists to understand its genetic diversity employing autosomal, Y-chromosomal and mitochondrial DNA markers. Y-chromosomal short tandem repeats (Y-STRs) are useful in understanding population substructures and reveal the patrilineal affinities among populations. Previous studies on Indian populations based on Y-STR markers were either limited to restricted number of markers or focused on few selected populations. In this study we genotyped 407 unrelated male individuals from 12 states in India employing the suite of Y-STRs present in PowerPlex Y23 (Promega, Madison, WI, USA). These populations clustered genetically close to each other irrespective of their geographic co-ordinates and were characterized primarily by R1a, H and L haplogroups. Interestingly, comparison with 129 worldwide populations showed genetic affinity of the Indian populations with few populations from Europe and Levantine. This study presents the first pan-Indian landscape of 23 Y-STRs and serves as a useful resource for construction of an Indian Y-STR database.

## Introduction

The male-specific human Y chromosome follows a strict mode of paternal inheritance and major portion comprises the non-recombining region (NRY). Y-chromosome is also widely acknowledged for its utility in providing one of the highest resolution tools for studying human population genetics owing to the aforementioned features of uniparental inheritance and non-recombining nature^1^. Y-chromosomal markers provide interesting insights into the past demographic events of a population as illustrated by several studies examining the patrilineal affinities among different world populations, including India^2–4^.

Contemporary human populations in India exhibit rich social, cultural and linguistic diversity. Multiple archeological, palaeoanthropological, linguistic and genetic studies highlight India as an interesting vault of ancient genetic pool^5,6^. It is believed that the genetic diversity exhibited by Indian populations may be because India along with other regions of South Asia served as an important corridor for ancient human migrations^7^. Genealogical studies have discerned that the Indian subcontinent was not only a recipient but also a donor of the genetic material to the world^6^. A report by Indian Genome Variation Consortium suggests that though India harbors higher genetic diversity compared to various worldwide populations, Indian populations exhibit low levels of genetic differentiation^8^. In the past, several studies have already addressed the genetic affinities among Indian populations employing autosomal markers^9,10^ and the uniparentaly inherited molecular markers located on mitochondria and Y-chromosome^11,12^.

Y-chromosomal studies in various populations of India show that the few Y-haplogroups (group of similar haplotypes derived from a common ancestor) were autochthonous in nature and dated back to the late Pleistocene epoch (e.g. haplogroups H, L1, F, C)^6,13^. However, few haplogroups (e.g. haplogroup J) that were reported to be frequent in India are also traced to outside of the Indian sub-continent^3^. Studying the Indian population history has always been a challenging task owing to its past demographic events and the complex organization of the extant human populations. Although, studies based on autosomal data reported a North to South genetic cline for non-tribal Indian populations^9,14^; other studies with Y-chromosomal markers were unable to corroborate such a cline^3,11,15^. Y-chromosome based worldwide comparisons of Indian populations showed their closest affinities with some European populations^3^.

In addition to playing an instrumental role in elucidating human population history; Y-STR markers were also widely used to study patrilineal diversity in various populations^16–18^. However, similar studies in Indian populations were confined to small restricted groups of India, which do not truly represent the genetic diversity possessed by them^17,19^. To the best of our knowledge, a comprehensive analyses representing populations from widely distributed geographical regions of India has not been attempted earlier employing 23 set of Y-STRs. In this study, we have genotyped 407 samples sourced from 12 different states (administrative provinces) representing North, South, East and West India using a multiplex of 23 Y-STR loci present in PowerPlex Y23 (PPY23) (Promega, Madison, WI, USA) system, to infer genetic relationship among the populations from different regions of the country. Our results show that these populations illustrated a closer genetic relationship among themselves, irrespective of their geographic distance. Moreover, while investigating the genetic relationship with populations from other regions of the world^20^, these populations interestingly showed greater genetic affinity with few populations from Europe and Levantine. Additionally, since this chemistry was reported to be forensically potent in various worldwide populations^20,21^; this panel was evaluated to gauge its forensic efficacy in Indian populations as well. This study apart from providing a comprehensive picture of Y-STR-based diversity of Indian populations, would also be of great utility for the development of a Y-STR database for forensic investigation purposes in India.

## Results and Discussion

### Molecular diversity

In order to obtain an estimate of molecular genetic diversity for the target STRs in Indian populations, we calculated genetic diversity (GD) values (a measure of the polymorphism at a locus) for each locus incorporated in the panel. After discarding four samples due to presence of more than one allele, we observed a total of 397 unique profiles with four haplotypes present in duplicates and one in triplicate which can be visualized through minimum spanning network (MSN) plot depicted in Supplementary Fig. S1. The GD for all the loci were exceeding 0.55, except for the DYS391 locus (Supplementary Fig. S2). Locus-wise analysis of molecular variance (AMOVA) showed that the majority of the genetic variation was observed within the populations (95.4%).

### Population-specific analysis

#### Y-STR allelic distribution among the target populations

To evaluate the distribution of Y-STR alleles, GD of the panel was calculated separately for each of the 12 populations. Higher values of GD (≥0.64) were observed for each of the populations indicating the polymorphic nature of the panel of markers (Supplementary Fig. S3) and would be highly informative for these populations. This was in agreement with the previous reports wherein this panel had shown high genetic diversity^17,19^. Even though the populations were sourced from 12 different geographic locations in India, this set of STRs in PPY23 system did not show much variation in allelic distribution across the populations (R_ST_~0.02).

#### Genetic relationship among populations

**Figure 1:**
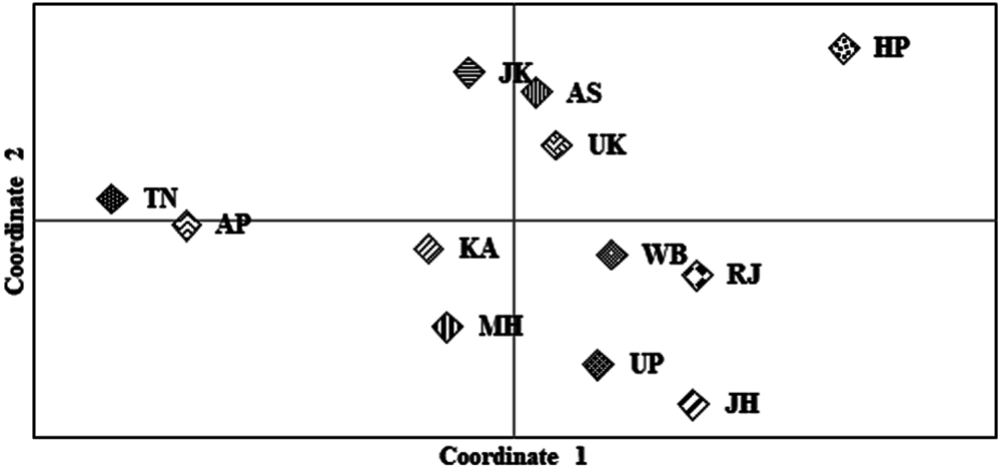
Plot to visualize PCoA based on genetic relationship between the populations from different bio-geographical regions of India. X and Y axes represent Coordinate 1 and 2, respectively and explain 39.42 and 26.32 percentage of total variance respectively. More than 60% of the variance was explained by the two coordinates. Each of the 12 populations occupied their relative position on the plot. As can be gleaned from the plot all the populations were observed to be in close proximity with each other irrespective of their geographic affiliations. The abbreviations are explained in Supplementary Table S1.

The principal coordinate analysis (PCoA) plot based on the pairwise Nei’s genetic distance among the 12 populations showed small genetic distance among them (Fig. 1). As can be seen from the plot, individuals from KA, MH, WB, RJ, UP and JH (the acronyms are expanded in Supplementary Table S1), although were sourced from geographically distant locations, clustered together in the plot. No significant correlation was observed between geography and the genetic distance (Fig. 1), an observation which corroborated our earlier findings based on autosomal STRs^22^. This was also supported by regression and correlation analysis based on pairwise geographic and genetic distance (R_ST_). Only 3.6% of the variation based on genetic distance could be explained by geographic distance, which was not statistically significant (p = 0.07). The correlation between geographic distance and genetic distance was also low (0.22) and statistically insignificant. Even after grouping the locations on the basis of geographic regions, none of the populations were found to be isolated as evident from the spatial analysis of AMOVA (SAMOVA), wherein only a small genetic variance was observed among the groups (F_CT_~0.02) (viz., North, West, East and South).

Similarly, discriminant analysis of principal components (DAPC) (Supplementary Fig. S4), which helps to visualize the differentiation between groups^23^ was used to assess the pattern of clustering in the Indian populations. It was observed that not only individuals belonging to the same geographic region were closely spaced in their individual cluster, but each of the 12 clusters were also closely overlapping and occupying central position in the plot. Thus, based on the 23 Y-STRs, only a subtle genetic variation was observed between populations. Previous studies had suggested a genetic cline from North to South for non-tribal populations in India^9^, however the same was not observed with the current set of Y-chromosome markers, which also corroborates the finding of Mondal *et al*.^3^.

#### Haplogroup studies

**Figure 2:**
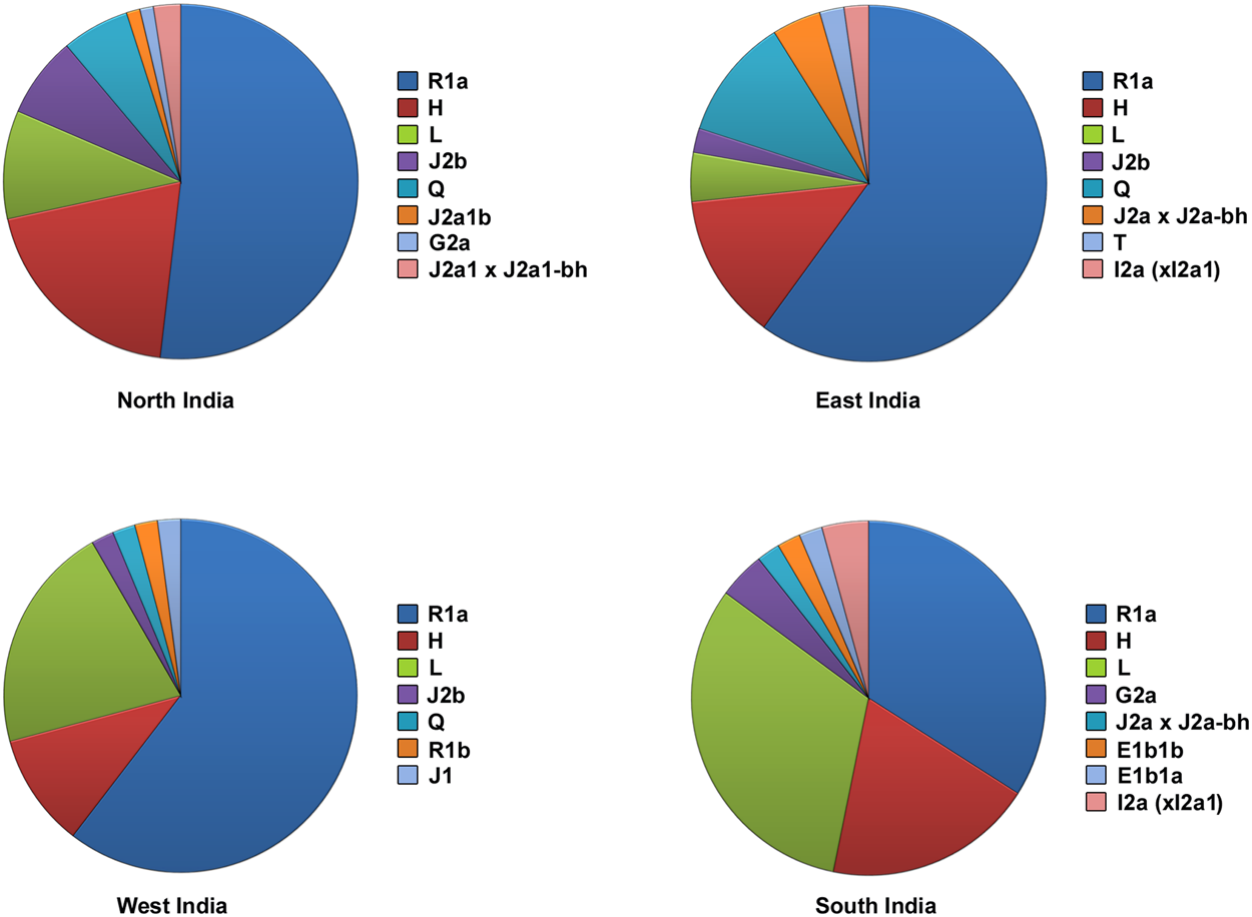
Haplogroup prediction using Whit Athey’s haplogroup predictor tool. Each of the pie chart represents proportion of haplogroups present in different biogeographic regions of the country i.e. North, West, South and East India. R1a, H and L haplogroups were found to be predominantly present in the country compared to the other haplogroups.

221 out of 357 individuals were assigned to haplogroups based on 23 Y-STRs using Whit-Athey’s algorithm. A total of 14 haplogroups (R1a, H, L, Q, J2b, J2a1 x J2a1-bh, J2a1b, J1, G2a, I2a (xI2a1), R1b, E1b1b, E1b1ba and T) were observed in this study, whereas 7 other haplogroups (viz., G2c, I1, I2a1, I2a (xI2a1), I2b1, J2a1h and N) mentioned in the haplogroup assigning tool were not observed in these samples. R1a (51.5%), H (16.2%) and L (15.8%) were the major haplogroups present throughout the country and accounted for more than three-fourths of the Y lineages. Figure 2 shows the proportion of haplogroups observed in North, West, South and East India. The abundance of these three haplogroups is also shown in the Network analysis (Supplementary Fig. S5) and their geographic distribution across the country is illustrated in Supplementary Fig. S6.

The occurrence of R1a was observed to decrease from north to south, while the haplogroup L displayed the opposite trend. In contrast to R1a and L haplogroups, haplogroup H was found to be distributed uniformly across the country. In a previous study, clades of R1a, J2, H and R2 were observed to be centered in North, Northwest, South and East India, respectively and R haplogroup and its clades were amongst the most frequent Y lineages found throughout India, East Europe and Central Asia^24^. R1a haplogroup is reported to be the most frequent haplogroup present in Eurasia^6,25^ and was also observed to be the most abundant in all the major geographic regions of India in this study.

The proportion of individuals representing haplogroup L was comparatively more (15.2%) in South India. Haplogroup L is thought to be associated with the expansion of farming^12^ and is known to be widely distributed in North Eurasia and in some Siberian groups^26^, with lower representation in populations of Europe, Middle East and East Asia^25^. Haplogroup H, which was suggested to be restricted mainly to Indian subcontinent^11,25^, was observed in 16.2% of individuals in the current study (Fig. 2). Considering the high diversity of haplogroup H in South Asia and its preponderance in India; the Indian subcontinent was proposed as the probable origin for this haplogroup^11,15^. The undifferentiated Q* haplogroup was reported at a low frequency in India and Pakistan^25^. In this study, haplogroup Q was present in approximately 5% of the samples but was absent in the populations from South India (Fig. 2).

Haplogroups J2b and J2a1 x J2a1-bh were the most prominent among the other branches of J2 in the Indian populations observed in the current study. The presence of J2 clades in India was suggested primarily due to the demic diffusion from West Asia^11,24^ whereas the occurrence of J1 haplogroup is believed to be rare^4^ and its presence was mainly attributed to the paternal gene pool contribution during Islamic expansion from Iranians and/or Arabians in the past^27^. Haplogroups J2a1b and J1 were represented by only one individual in the present study. G2a, another rare haplogroup was observed in only 3 individuals (1.3%) and was absent in individuals from western and eastern parts of India. Since these haplogroups were based on predictor algorithm, caution has to be exercised while drawing interpretations based on these tools. Nevertheless, the use of large number of STRs (>17 loci), which was the case in this work, is able to provide a reasonable estimate of haplogroup prediction^28^, as is being supported by earlier studies based on Y-chromosomal STRs^29^.

#### Comparison with other populations

Our study compared populations representing four geographic regions from India, with 129 populations across the world which were reported previously^20^. The multidimensional scaling (MDS) plot (Supplementary Fig. S7, abbreviations explained in Supplementary Table S2) and its magnified portion (for ease of readability) (Supplementary Fig. S8) portray the genetic affinities of populations examined in this study with the 129 populations worldwide. We observed that the Indian populations in this study were in close proximity to the other Indian populations studied in the past (Gujarati Indians in Texas, Indians in Singapore, South-Indian (Tamils)). We also observed few other populations to be genetically closer to Indian populations viz., Italy (Calabria), Barnaya-Hungary (Romani), London-UK (British-Asians), Lebanon, Iraq, Bolivia (Mestizo), Panama, Hungary (Budapest), Bolivia (Native-Americans), Estonia, Latvia and Lithuania (Vilnius).

Our findings are in agreement with previous anthropological and linguistic reports supporting genetic similarity of Romanis and Indians^30,31^. In fact, the north-western region of India is believed to be the most probable region of origin of Romanis^32,33^. Previous genome-wide scans revealed that the Romanis are genetically close to Indians^34^, whereas the genetic closeness of Indians and British-Asians from London-UK can be attributed to the common ethnicity of the samples in these two populations. As the Levant countries are strategically located at the cross-roads of Africa, Eurasia and South Asia, they might have witnessed the ancient migration of humans out of Africa^35^. The genetic affinity of populations belonging to Lebanon and Iraq from the Levant region to Indian populations may be attributed to their geographic proximity, which is also supported by an earlier study^35^, wherein the authors had used Y-chromosome haplotypes as molecular tools to reveal relationship between populations of Arabian peninsula and South Asia. The populations from India in this study interestingly also showed proximity to the populations from Italy, Panama, Bolivia (Native Americans) and Baltic (Estonia, Latvia and Lithuania) populations as well, which needs further investigations involving Y-SNPs and other ancestry informative markers to better understand the significance of this finding.

To examine the genetic relationship of the populations tested in the current study with the Indian populations reported previously in Y Chromosome Haplotype Reference Database (YHRD), MDS analysis was performed, which showed very low genetic distance between them. Further, R_ST_ based genetic relationship with other neighboring as well as few European countries was visualized in the heatmap presented in Supplementary Fig. S9. In agreement with previous observations and expectedly, the Indian populations from this study were closer to the other Indian populations studied previously as well as populations from Bangladesh and Pakistan. The genetic affinities of Indian populations with few European populations could be attributed to the higher mutation rates of the STRs (that is further accentuated by the inclusion of two rapidly mutating Y-STRs in the PPY23 system), which might have resulted in similar distribution of Y-STR alleles in different populations as a result of identity-by-state (IBS) rather than being inherited from a common ancestor following identity-by-descent (IBD)^18,36^.

#### Forensic applicability in Indian populations

On a different perspective, the utility of the 23 Y-STR chemistry for its forensic applicability in India populations was also investigated. High haplotype diversity (HD) (0.9948793876) and discrimination capacity (DC) (0.98511166253) values demonstrated its informativeness in the Indian populations. As expected, this panel exhibited a low combined match probability (CMP) (0.00264578799), which was comparable to the previously published reports^17,19^.

Allelic distribution of the six newly incorporated Y-STRs, which were introduced in PPY23 system and were not part of the previous panel of 17 Y-STRs (AmpF*l*STR Yfiler, Thermo Fisher Scientific, Waltham, USA) are shown in Supplementary Fig. S10. The DYS481 locus showed the highest number of alleles and microvariants but the distribution of alleles was not uniform. The DYS549 locus was observed to be the least polymorphic marker among all the six newly incorporated STRs. DYS570 and DYS576 loci showed greater polymorphism with a more uniform distribution of alleles, which make them highly informative.

## Conclusion

Here, we report a detailed Y-STR analysis of Indian samples contributing to enlarge the knowledge on the genetic landscape of India. PCoA, SAMOVA and DAPC analysis substantiated a closer genetic affinity among the populations in India. Though Athey’s algorithm was reported to be efficient for prediction of haplogroups as compared to other available tools^37^, SNP-based analysis would increase the resolution and accuracy of the predicted haplogroup assignment. In agreement with previous studies, a higher proportion of R1a, H and L haplogroups was observed in Indian populations. The data in this study deepens the panorama of Y-chromosomal diversity in Indian populations, and will be useful for comparison with other populations in future studies. The Y-STR panel was found to be equally efficient in all the populations and can be employed irrespective of their geography in India. The data obtained from this study would be of great use for statistical calculations of random match frequency estimates for forensic case-work analysis. This study represents the first comprehensive analysis of Indian populations with 23 Y-STRs, turning available data of great interest not only to the broad field of population genetics but also to the community of forensic geneticists.

## Materials and Methods

### Sample collection and DNA isolation

After receiving the written informed consent from 407 unrelated adult male volunteers from 12 different states belonging to four major geographical regions of India, saliva samples were collected in an unstimulated fashion in sterile tubes containing 2 mL of lysis buffer as described previously^22,38^. The geographical locations from where the samples were collected are shown in Supplementary Fig. S11 and Table S1. This study was approved by the Institutional Bioethics Committee of the Centre for DNA Fingerprinting and Diagnostics (CDFD) and all the protocols pertaining to sample collection were according to the approved guidelines. The saliva sample tubes were sealed and transported to the laboratory at room temperature for DNA extraction using the salt precipitation method as described previously^22,38^.

### Y-STR markers and genotyping protocols

Individuals were genotyped for 23 Y-STR markers incorporated in PowerPlex Y23 (PPY23) system (Promega, Madison, WI, USA). The Y-STRs were amplified according to the manufacturer’s instructions in a GeneAmp 9700 thermal cycler (Thermo Fisher Scientific, Waltham, USA) followed by capillary electrophoresis on the ABI Prism 3130*xl* Genetic Analyzer (Thermo Fisher Scientific) and data was analyzed using the GeneMapper ID version 3.2.1 (Thermo Fisher Scientific). The control DNA 2800M was genotyped for quality control purposes. A certificate for quality assurance was obtained from the Department of Forensic Genetics at the Charite´–Universitätsmedizin, Berlin, Germany, which is a mandatory criterion for submitting the Y-STR data to the YHRD. The data was submitted to YHRD and 12 accession numbers were obtained, which are mentioned in Supplementary Table S1.

### Statistical analyses

Four samples that were found to be biallelic at few loci were discarded and further analyses were performed on the remaining 403 samples. Allele and haplotype frequencies were calculated by counting method. GD (GD = $\frac{n}{(n-1)}(1-\sum _{i}{p}_{i}^{2})$, where n is the total number of samples and *p*_*i*_ is the frequency of *i-th* allele) and HD (HD = $\frac{n}{(n-1)}(1-\sum _{i}\,{{h}_{i}}^{2})$, where *h*_*i*_ is the haplotype frequency) were calculated as per previous reports^2,20^. MSN was generated using *poppr*^39^ package implemented in R v3.1.2 to visualize the number of unique and redundant haplotypes. Arlequin^40^ was used to perform locus-wise AMOVA. For comparison of the genetic distance among the current populations as well as with that of the other populations reported in YHRD, DYS385a/b locus and the samples with micro-variants were omitted. GenALEx v6.5^41,42^ was used to assess the molecular diversity of the Y-STR panel and to test the performance of the panel in each population. PCoA (based on the pairwise Nei’s genetic distance), to visualize genetic relationship among the populations was performed using GenALEx. *Poppr* package in R v3.1.2 was also used to perform DAPC.

AMOVA based on R_ST_ and MDS was performed using YHRD-based online tool (http://www.yhrd.org) to infer the genetic relationship among populations. To test whether there was any association between the genetic and geographic distances among these populations, SAMOVA was performed^43^. R_ST_ values derived from pairwise comparison with other populations of the world were also inferred employing the YHRD tool. To investigate if the population substructure affects the Y-STR distribution, the geographic locations were considered as its representative and regression analysis was performed using the geographic distance between sampling locations as a covariate for the genetic distance (pairwise R_ST_). The correlation between the geographic distance and the R_ST_ across all pairs of populations was also tested using R v3.1.2. Further, MDS plots were drawn using the sammon function from MASS package implemented in R v3.1.2 to visualize the genetic relationship between the populations in the current study with those reported earlier. Individuals were assigned respective haplogroups derived from Y-STRs using Whit Athey’s haplogroup predictor^44^. As recommended in the instructions for users in the Whit Athey’s haplogroup predictor tool’s website, minimum score and minimum probability to assign an individual to a particular haplogroup was set to 40 and 95 percent, respectively.

Median joining (MJ) networks for Y-STR haplotypes within specific Y-haplogroups were constructed using the software NETWORK 5.0.0.1^45^. The network analyses were carried out for the three most abundant lineages observed in this study. The variance at each locus for the haplogroups was calculated in the whole dataset using R v3.1.2. The weights assigned to each locus were inversely proportional to the observed variance as reported previously^46^. Briefly, weights for a range of variance were assigned as follows: for variance 0.0–0.2, weight = 10; for variance 0.2–0.4, weight = 8; for variance 0.4–0.6, weight = 6; for variance 0.6–0.8, weight = 4 and for variance >0.8, weight = 2. To view the spatial distribution of the major haplogroups, Surfer15 of Golden Software (Golden Software Inc., Golden, Colorado) was used to generate iso-frequency maps. To investigate the forensic applicability of the multiplex in the Indian populations, the sum of squared haplotype frequencies was calculated to obtain CMP of the panel of markers. DC i.e. the ratio between the number of unique haplotypes to the total number of haplotypes was calculated.

## Electronic Supplementary Material

Supplementary Information
