# Supplementary material for: A comprehensive portrait of Y-STR diversity of Indian populations and comparison with 129 worldwide populations
Source: Sci Rep. 2018 Oct 18;8:15421. doi: 10.1038/s41598-018-33714-2 (PMC6194109; doi:10.1038/s41598-018-33714-2)
Supplement: Supplementary file 1 — Supplementary Information [file 41598_2018_33714_MOESM1_ESM.pdf]

**Title:** A comprehensive portrait of Y-STR diversity of Indian populations and comparison with 129 worldwide populations

**Authors:** Mugdha Singh[a, b], Anujit Sarkar[a] and Madhusudan R. Nandineni[*a, c]

[a]Laboratory of Genomics and Profiling Applications, Centre for DNA Fingerprinting and Diagnostics, Uppal, Hyderabad, Telangana State, India, 500039.

[b]Graduate studies, Manipal Academy of Higher Education, Manipal, Karnataka, India.

[c]Laboratory of DNA Fingerprinting Services, Centre for DNA Fingerprinting and Diagnostics, Uppal, Hyderabad, Telangana State, India, 500039.

Tel.: +91-40-2721-6141
Fax: +91-40-2721-6006
*Corresponding author
E-mail: nandineni@cdfd.org.in

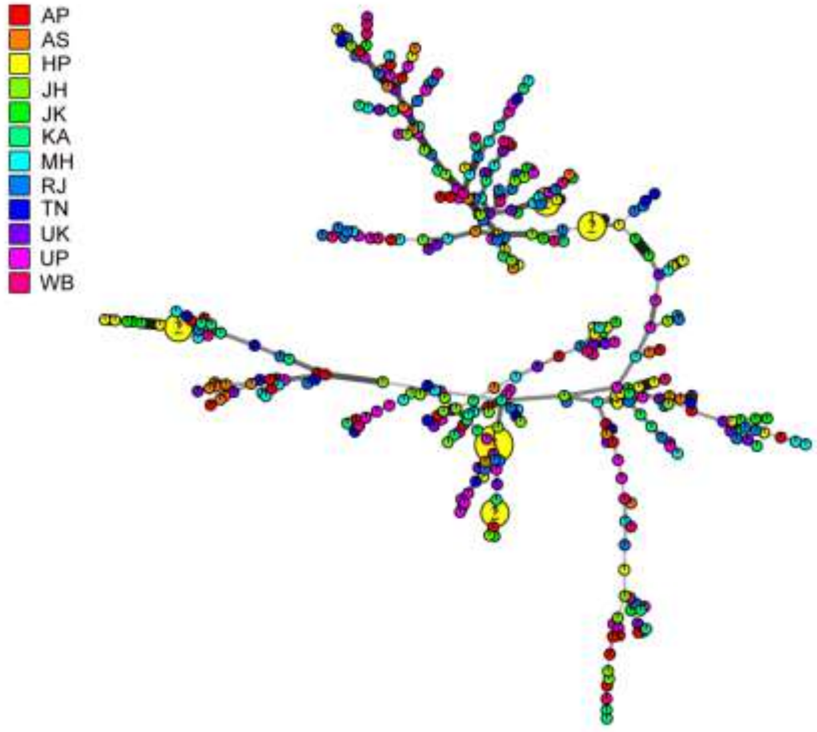


**Supplementary Fig. S1:** Minimum spanning network, wherein each circle represent a haplotype. Size of the circle is proportional to the number of haplotype. Each of the sampling location is depicted by a different colour. All the abbreviations are explained in supplementary Table S1.

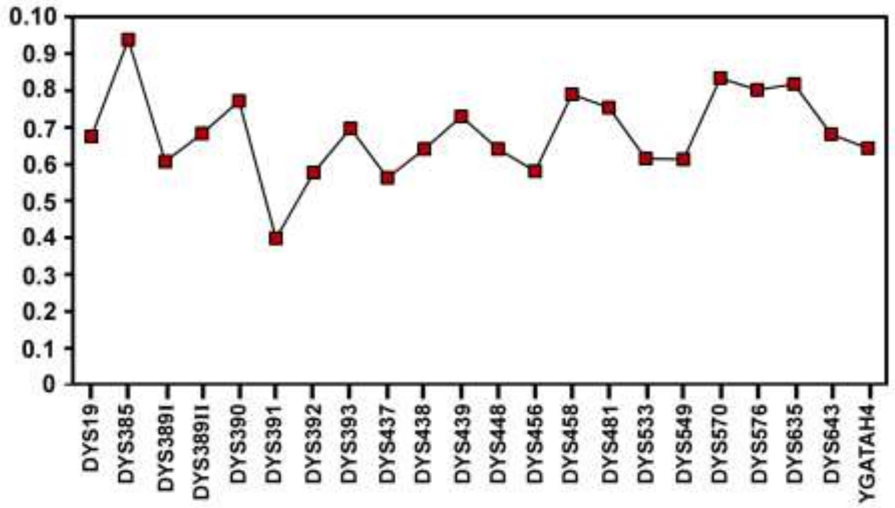


**Supplementary Fig. S2:** Genetic diversity (GD) across different loci. X axis represent Y-STR loci; Y axis represents the corresponding values of GD. All the values obtained were higher than 0.55 except for DYS391 locus.

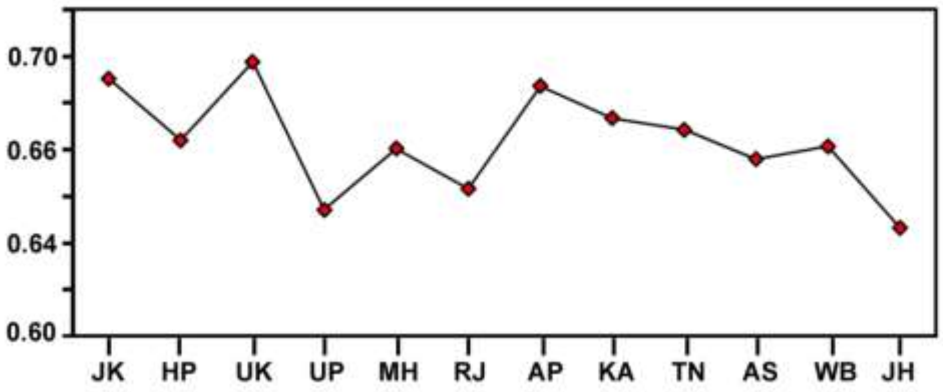


**Supplementary Fig. S3:** GD across sub-populations. X axis represents different populations across India; Y axis represents their corresponding GD values. Values were above 0.64 for all the studied sub-populations. All abbreviations are explained in supplementary Table S1.

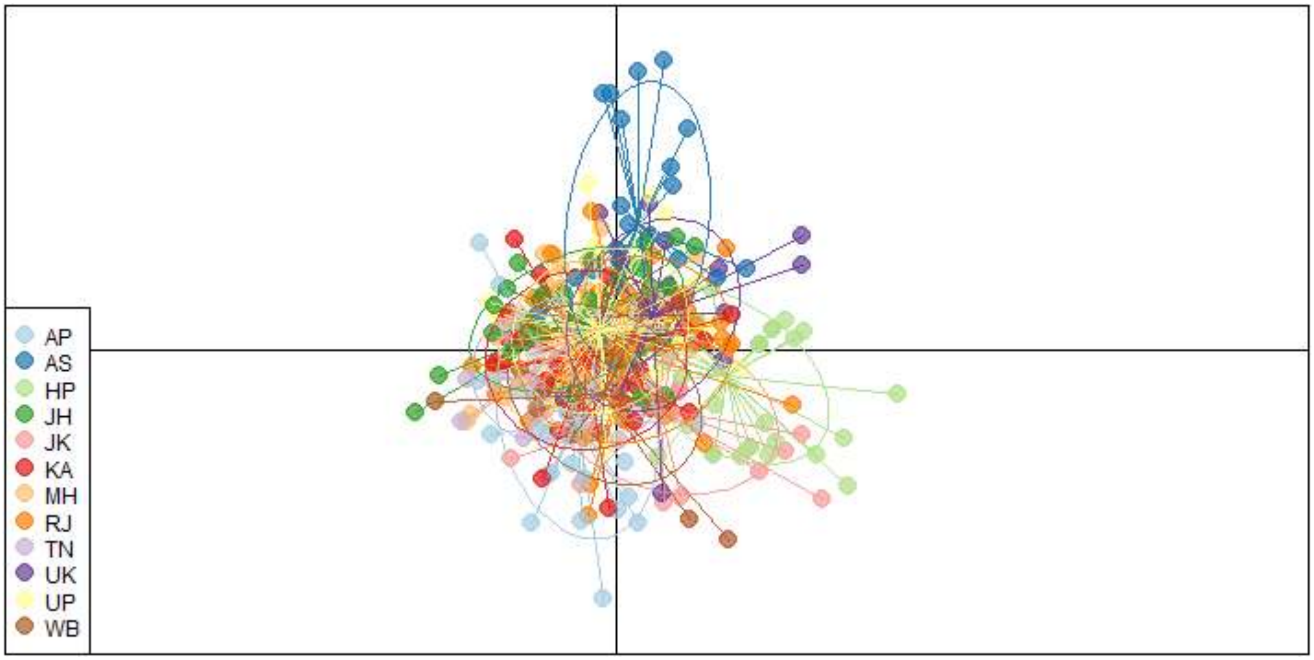


**Supplementary Fig. S4:** Discriminant analysis of principle components (DAPC) to assess contribution of individuals towards any clustering if present. Circles in the plot represent different individuals and the colors filled in the circle denote the population affiliation. The color key representing different sampling site is at the left bottom of the plot. As can be seen in the plot; there is one cluster and the populations are not significantly genetically distant. All abbreviations are explained in supplementary Table S1.

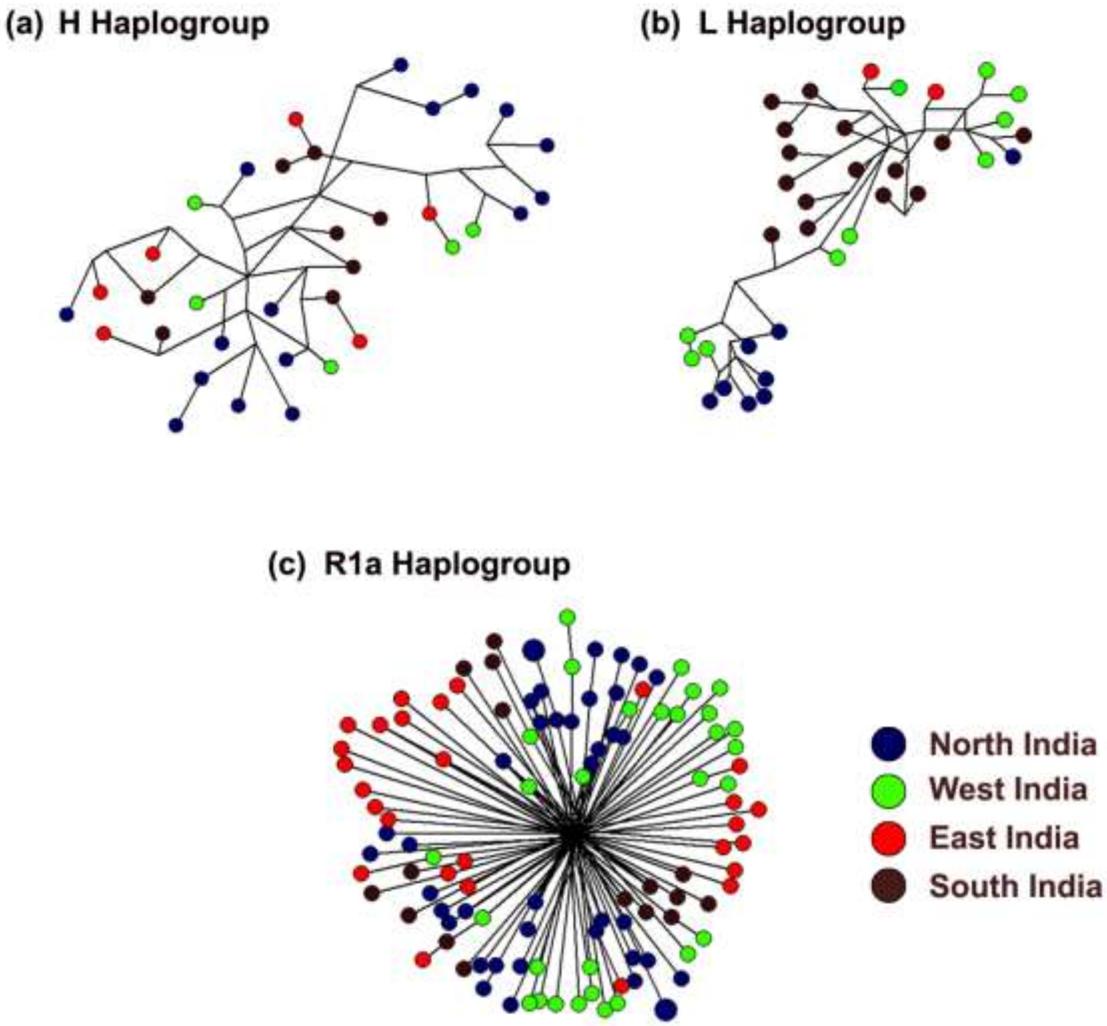


**Supplementary Fig. S5:** Median-joining network based on distribution of Y-STR haplotypes in the Indian populations for three haplogroups (a) H, (b) L, and (c) R1a. The colour codes have been assigned based on the geographic region of the samples.

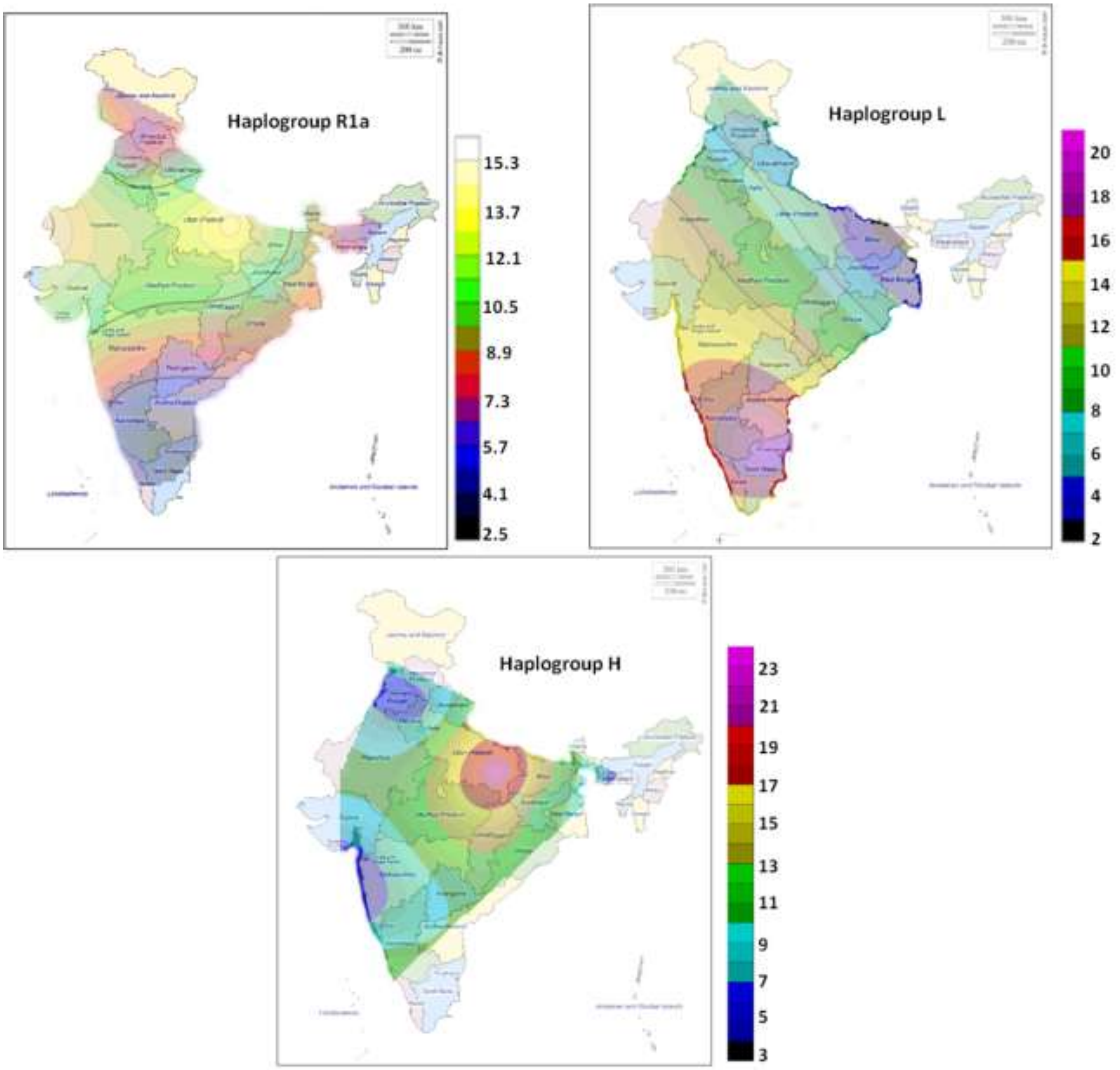


**Supplementary Fig. S6:** Contour maps showing the distribution of major haplogroups in Indian populations based on frequency of R1a (a), L (b) and H (c). The color scale indicates the frequency of the haplogroup at sampling locations.

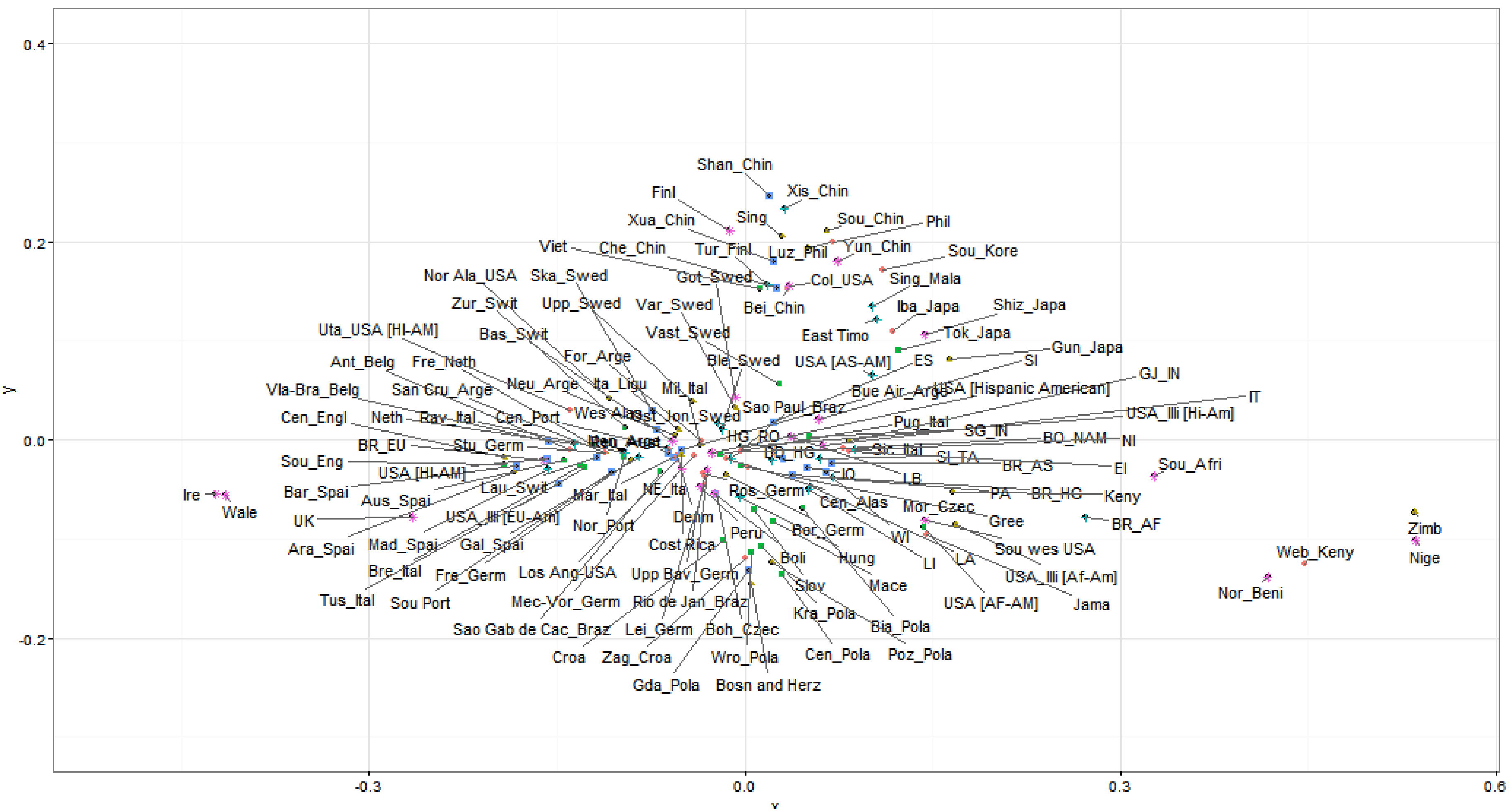


**Supplementary Fig. S7:** MDS plot to view the patrilineal relationships/affinities. The populations in the current study were found to be closer to Indian populations sampled in other studies. Abbreviations of the names of populations are provided in the supplementary Table S2. A zoomed picture of populations which are relatively much closer than other populations analyzed is shown in Fig. S8.

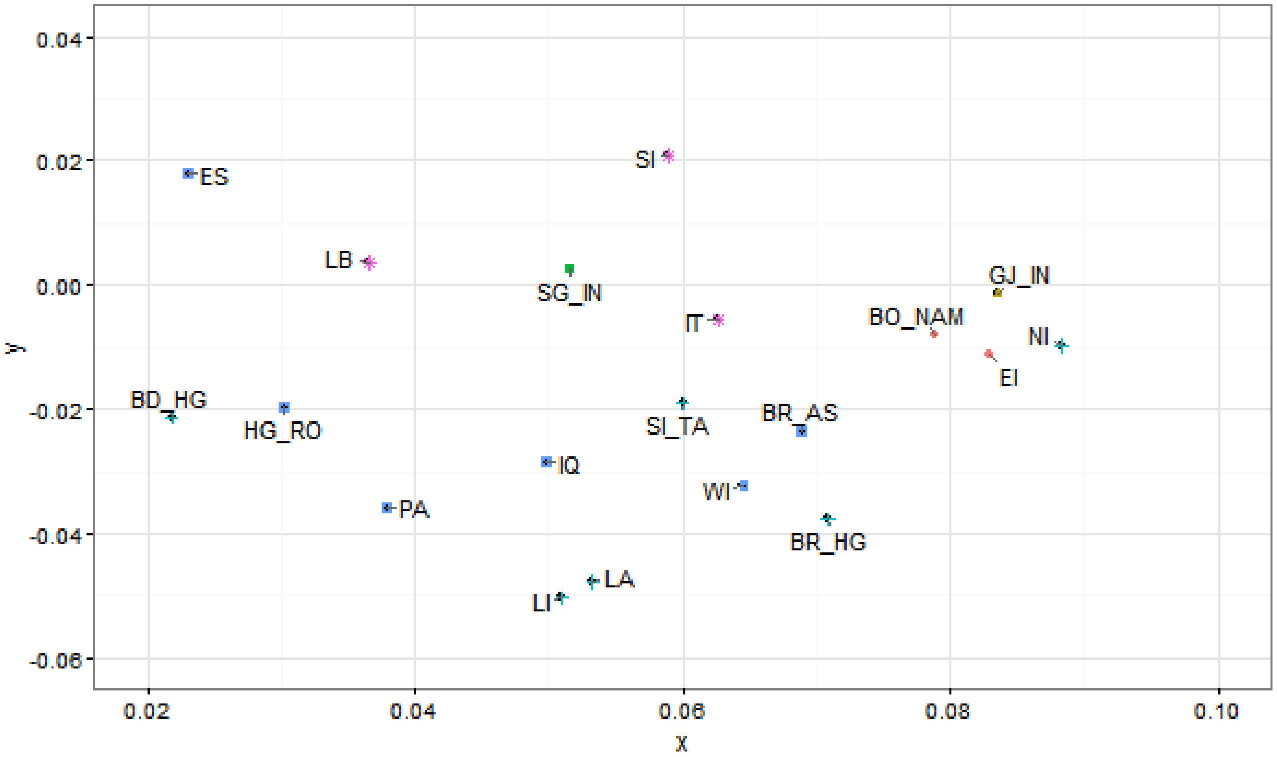


**Supplementary Fig. S8:** Enlarged view of the MDS plot to view the patrilineal relationships/affinities. The Indian populations from the present study (NI, SI, EI and WI) clustered in close vicinity to other populations from India previously studied viz., GJ_IN (Gujarati Indians in Texas), SG_IN (Indians in Singapore), SI_TA (Tamils speakers from South India). Signature of close patrilineal genetic affinity with HG_RO, BR_HG, BD_HG (populations from Hungary), BO_NAM (Native Americans from Bolivia), PA (Panama), IQ (Iraq), LB (Lebanon), LI (Lithuania), LA (Latvia), ES (Estonia), IT (Italy) and BR_AS (British Asians from London-UK) was also observed. All abbreviations are as described in supplementary Table S2.

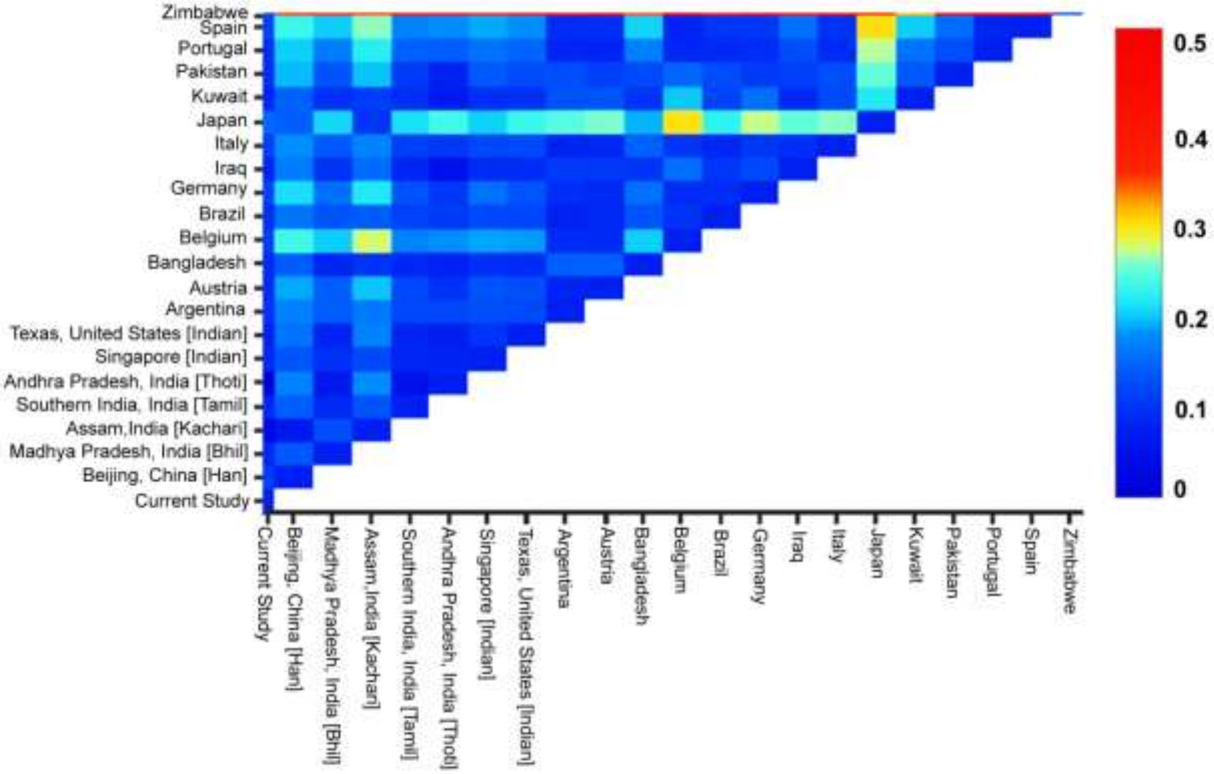


**Supplementary Fig. S9:** Heat map showing $R_{ST}$ based genetic relationship between populations in current study and other neighboring populations. Blue to red color bar indicates increase in genetic distance. The populations tested in this study were found to be in close genetic relationship with other populations from India studied previously.

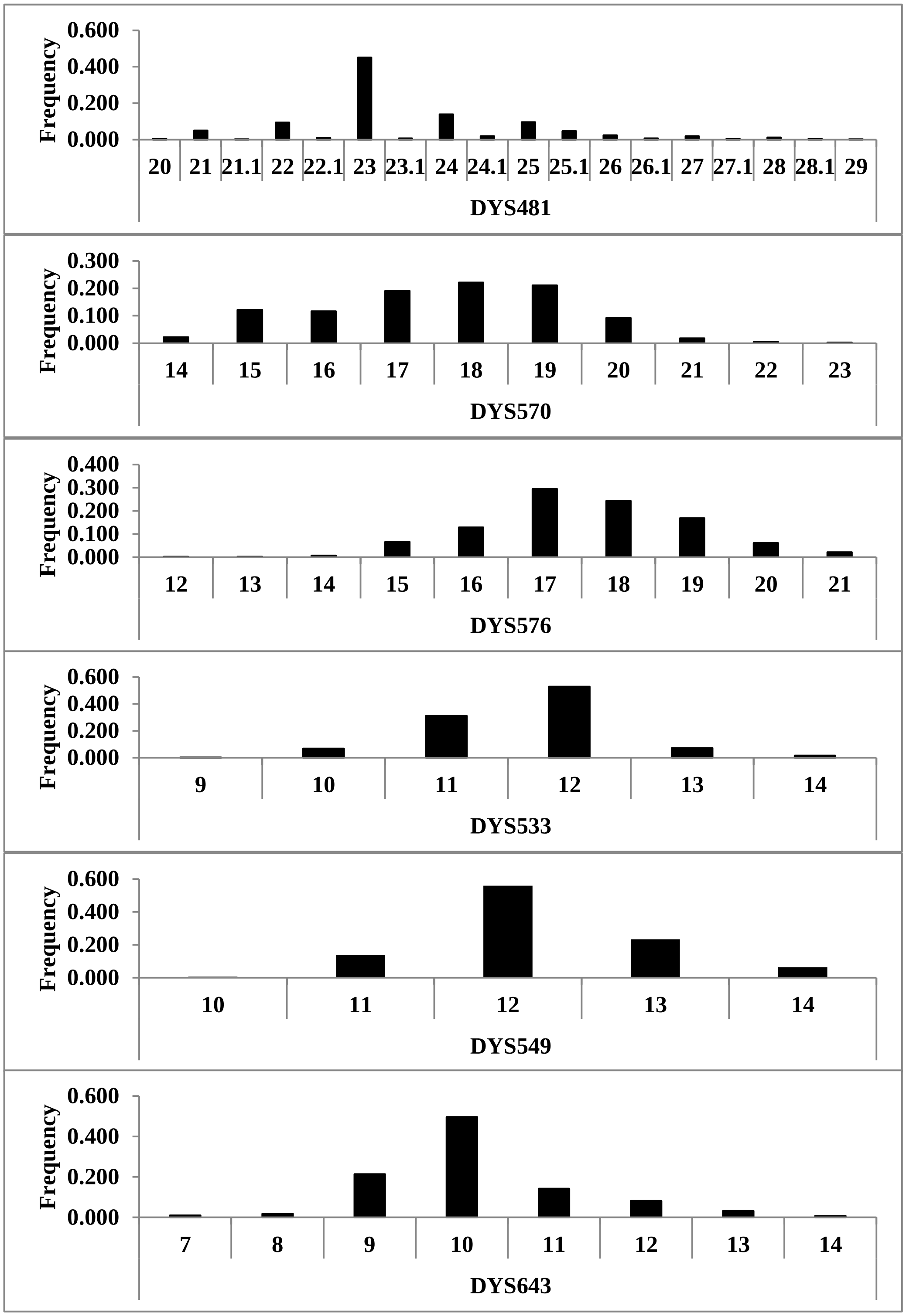


**Supplementary Fig. S10:** Allelic distribution of the six newly incorporated Y-STRs. Each of the six graphs represents the allelic distribution of the recently introduced Y-STRs. X axis of each graph represents different alleles and Y axis represents their corresponding allele frequencies. The highest and the least number of alleles were observed for DYS481 and DYS549 loci respectively. DYS570 locus was observed to be the most informative locus and showed the most uniform distribution of alleles when compared to the other above mentioned Y-STRs.

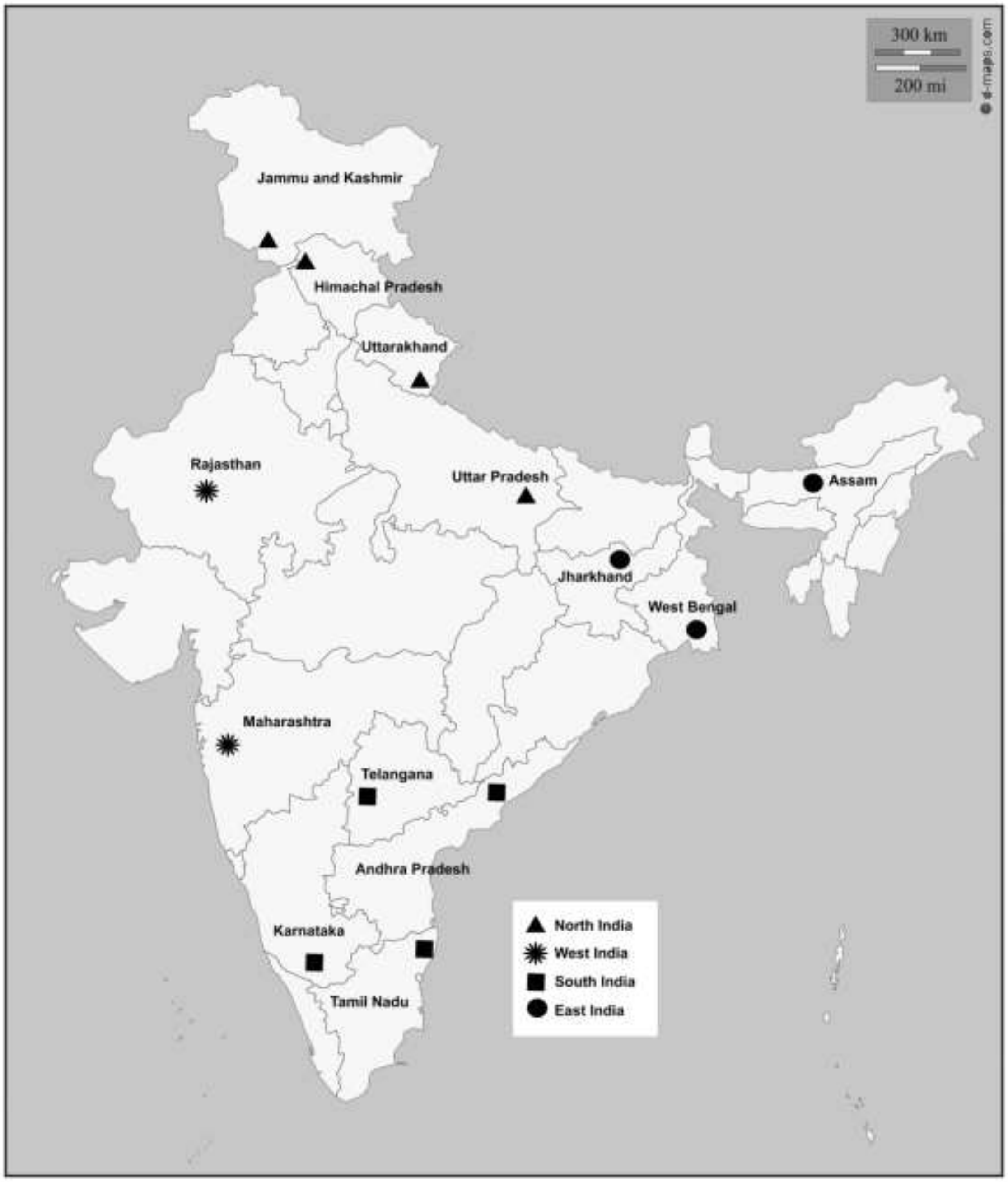


**Supplementary Fig. S11:** Distribution of sampling sites is shown on map of India (adapted from d-maps.com, https://d-maps.com/carte.php?num_car=24852&lang=en) with administrative boundaries (States). The sample collection represents North, West, South and East biogeographic regions of India. Details of sample size (n), sampling sites and the corresponding biogeographic regions are given in supplementary Table S1. The samples from Telangana and Andhra Pradesh were clubbed and collectively analyzed as one population (i.e. Andhra Pradesh).

**Table S1:** Biogeographic region, sampling location and the sample size (n) corresponding to the sampling location. The corresponding abbreviations for biogeographic region and the sampling location were used in other analyses in this study. *The samples from Telangana and Andhra Pradesh were combined and analyzed as one population (i.e. Andhra Pradesh).

| Sl. No. | Biogeographic region | Sampling location and abbreviation | Number of individuals sampled from each location (n) | YHRD accession Number |
|---|---|---|---|---|
| 1 | North India (NI) | Jammu and Kashmir (JK) | 26 | YA004336 |
| 2 | North India (NI) | Himachal Pradesh (HP) | 40 | YA004335 |
| 3 | North India (NI) | Uttarakhand (UK) | 32 | YA004343 |
| 4 | North India (NI) | Uttar Pradesh (UP) | 58 | YA004342 |
| 5 | West India (WI) | Rajasthan (RJ) | 46 | YA004340 |
| 6 | West India (WI) | Maharashtra (MH) | 36 | YA004339 |
| 7 | South India (SI) | Andhra Pradesh (AP)* | 35 | YA004333 |
| 8 | South India (SI) | Karnataka (KA) | 37 | YA004338 |
| 9 | South India (SI) | Tamil Nadu (TN) | 18 | YA004341 |
| 10 | East India (EI) | Assam (AS) | 25 | YA004334 |
| 11 | East India (EI) | West Bengal (WB) | 23 | YA004344 |
| 12 | East India (EI) | Jharkhand (JH) | 31 | YA004337 |

| **Table S2** Abbreviation and expansion of the names of the populations used for studying the affinities with Indian populations | | |
|---|---|---|
| Sl. No. | Abbreviation of populations | Expansion of abbreviations used |
| 1 | Ant_Belg | Antwerpen, Belgium [Belgian] |
| 2 | Ara_Spai | Aragon, Spain [Spanish] |
| 3 | Aus_Spai | Asturias, Spain [Spanish] |
| 4 | Bar_Hung | Baranya, Hungary [Romani] |
| 5 | Bar_Spai | Barcelona, Spain [Spanish] |
| 6 | Bas_Swit | Basel, Switzerland [Swiss] |
| 7 | Bei_Chin | Beijing, China [Han] |
| 8 | Ber_Germ | Berlin, Germany [German] |
| 9 | Bia_Pola | Bialystok, Poland [Polish] |
| 10 | Ble_Swed | Blekinge, Sweden [Swedish] |
| 11 | Boh_Czec | Bohemia, Czechia [Czech] |
| 12 | Boli | Bolivia [Mestizo] |
| 13 | Boli_Nati Amer | Bolivia [Native American] |
| 14 | Bosn and Herz | Bosnia and Herzegovina [Bosnian] |
| 15 | Bre_Ital | Brescia, Italy [Italian] |
| 16 | Bud_Hung | Budapest, Hungary [Hungarian] |
| 17 | Bue Air_Arge | Buenos Aires, Argentina [European] |
| 18 | Cal_Ital | Calabria, Italy [Italian] |
| 19 | Cen_Alas | Central Alaska, USA [Athapaskan] |
| 20 | Cen_Engl | Central England [English] |
| 21 | Cen_Pola | Central Poland, Poland [Polish] |
| 22 | Cen_Port | Central Portugal, Portugal [Portuguese] |
| 23 | Che_Chin | Chengdu, China [Han] |
| 24 | Col_USA | Colorado, USA [Han] |
| 25 | Denm | Copenhagen, Denmark [Danish] |
| 26 | Cost Rica | Costa Rica [Mestizo] |
| 27 | Croa | Croatia [Croatian] |
| 28 | East Timo | East Timor [Melanesian] |
| 29 | Esto | Estonia [Estonian] |
| 30 | Finl | Finland [Finnish] |
| 31 | For_Arge | Formosa, Argentina [European] |
| 32 | Fre_Germ | Freiburg, Germany [German] |
| 33 | Fre_Neth | Friesland, Netherlands [Dutch] |
| 34 | Gal_Spai | Galicia, Spain [Spanish] |
| 35 | Gda_Pola | Gdansk, Poland [Polish] |
| 36 | Got_Swed | Gotland, Sweden [Swedish] |
| 37 | Gree | Greece [Greek] |
| 38 | Gun_Japa | Gunma, Japan [Japanese] |

| | | |
|---|---|---|
| 39 | Viet | Ho Chi Minh City, Vietnam [Kinh] |
| 40 | Hung | Hungary [Hungarian] |
| 41 | Hung_Roma | Hungary [Romani] |
| 42 | Nige | Ibadan, Nigeria [Yoruba] |
| 43 | Iba_Japa | Ibaraki, Japan [Japanese] |
| 44 | USA_Illi [Af-Am] | Illinois, USA [African American] |
| 45 | USA_Illi [EU-Am] | Illinois, USA [European American] |
| 46 | USA_Illi [Hi-Am] | Illinois, USA [Hispanic American] |
| 47 | Ira | Iraq [Iraqi] |
| 48 | Ire | Ireland [Irish] |
| 49 | Jama | Jamaica [Jamaican] |
| 50 | Keny | Kinyawa, Kenya [Maasai] |
| 51 | Kra_Pola | Krakow, Poland [Polish] |
| 52 | Latv | Latvia [Latvian] |
| 53 | Lau_Swit | Lausanne, Switzerland [Swiss] |
| 54 | Leba | Lebanon [Lebanese] |
| 55 | Lei_Germ | Leipzig, Germany [German] |
| 56 | Ita_Ligu | Liguria, Italy [Italian] |
| 57 | Slov | Ljubljana, Slovenia [Slovenian] |
| 58 | Lon_UK [BR-AF] | London, UK [British African] |
| 59 | Lon_UK [BR-AS] | London, UK [British Asian] |
| 60 | Lon_UK [BR-EU] | London, UK [British European] |
| 61 | Los Ang-USA | Los Angeles, USA [Mexican] |
| 62 | Luz_Phil | Luzon, Philippines [Filipino] |
| 63 | Mace | Macedonia [Macedonian] |
| 64 | Mad_Spai | Madrid, Spain [Spanish] |
| 65 | Mar_Ital | Marche, Italy [Italian] |
| 66 | Mec-Vor_Germ | Mecklenburg-Vorpommern, Germany [German] |
| 67 | Men_Arge | Mendoza, Argentina [European] |
| 68 | Mil_Ital | Milano, Italy [Italian] |
| 69 | Mor_Czec | Moravia, Czechia [Czech] |
| 70 | Neth | Netherlands [Dutch] |
| 71 | Neu_Arge | Neuquen, Argentina [European] |
| 72 | Nor_Beni | North-Benin, Benin [Beninese] |
| 73 | NE_Ita | Northeastern Italy, Italy [Italian] |
| 74 | Nor Ala_USA | Northern Alaska, USA [Inupiat] |
| 75 | Nor_Port | Northern Portugal, Portugal [Portuguese] |
| 76 | Ost_Jon_Swed | Östergötland/Jönköping, Sweden [Swedish] |
| 77 | Pana | Panama [Panamanian] |
| 78 | Peru | Peru [Peruvian] |
| 79 | Phil | Philippines [Filipino] |

| | | |
|---|---|---|
| 80 | Poz_Pola | Poznan, Poland [Polish] |
| 81 | Pug_Ital | Puglia, Italy [Italian] |
| 82 | Rav_Ital | Ravenna, Italy [Italian] |
| 83 | Reu_Aust | Reutte, Austria [Tyrolean] |
| 84 | Rio de Jan_Braz | Rio de Janeiro, Brazil [Admixed Brazilian] |
| 85 | Ros_Germ | Rostock, Germany [German] |
| 86 | San Cru_Arge | Santa Cruz, Argentina [European] |
| 87 | Sao Gab de Cac_Braz | Sao Gabriel de Cachoeira, Brazil [Native American] |
| 88 | Sao Paul_Braz | Sao Paulo, Brazil [Admixed Brazilian] |
| 89 | Shan_Chin | Shantou, China [Minnan Han] |
| 90 | Shiz_Japa | Shizuoka, Japan [Japanese] |
| 91 | Sic_Ital | Sicily, Italy [Italian] |
| 92 | Sing | Singapore [Han] |
| 93 | Sing_Indi | Singapore [Indian] |
| 94 | Sing_Mala | Singapore [Malay] |
| 95 | Ska_Swed | Skaraborg, Sweden [Swedish] |
| 96 | Sou_Afri | South Africa [Xhosa] |
| 97 | Sou_Kore | South Korea [Korean] |
| 98 | Sou_Chin | Southern China, China [Han] |
| 99 | Sou_Eng | Southern England [English] |
| 100 | Sou_Ind [Tam] | Southern India, India [Tamil] |
| 101 | Sou Port | Southern Portugal, Portugal [Portuguese] |
| 102 | Sou wes USA | Southwestern USA, USA [African American] |
| 103 | Stu_Germ | Stuttgart, Germany [German] |
| 104 | Tex_USA [GJ-IN] | Texas, USA [Gujarati Indians] |
| 105 | Tok_Japa | Tokyo, Japan [Japanese] |
| 106 | Tur_Finl | Turku, Finland [Finnish] |
| 107 | Tus_Ital | Tuscany, Italy [Italian] |
| 108 | UK | United Kingdom [British] |
| 109 | Upp Bav_Germ | Upper Bavaria, Germany [German] |
| 110 | Upp_Swed | Uppsala, Sweden [Swedish] |
| 111 | USA [AF-AM] | USA [African American] |
| 112 | USA [AS-AM] | USA [Asian American] |
| 113 | USA [HI-AM] | USA [European American] |
| 114 | USA [Hispanic American] | USA [Hispanic American] |
| 115 | Uta_USA [HI-AM] | Utah, USA [European American] |
| 116 | Var_Swed | Värmland, Sweden [Swedish] |
| 117 | Vast_Swed | Västerbotten, Sweden [Swedish] |
| 118 | Vil_Lith | Vilnius, Lithuania [Lithuanian] |
| 119 | Vla-Bra_Belg | Vlaams-Brabant, Belgium [Belgian] |
| 120 | Wale | Wales [Welsh] |

| | | |
|---|---|---|
| 121 | Web_Keny | Webuye, Kenya [Luhya] |
| 122 | Wes Alas | Western Alaska, USA [Yupik] |
| 123 | Wro_Pola | Wroclaw, Poland [Polish] |
| 124 | Xis_Chin | Xishuangbanna, China [Dai] |
| 125 | Xua_Chin | Xuanwei, China [Han] |
| 126 | Yun_Chin | Yunnan, China [Bai] |
| 127 | Zag_Croa | Zagreb, Croatia [Croatian] |
| 128 | Zimb | Zimbabwe [Zimbabwean] |
| 129 | Zur_Swit | Zürich, Switzerland [Swiss] |
| 130 | NI_IND | North India |
| 131 | WI_IND | West India |
| 132 | SI_IND | South India |
| 133 | EI_IND | East India |
